# Virological response, HIV-1 drug resistance mutations and genetic diversity among patients on first-line antiretroviral therapy in N’Djamena, Chad: findings from a cross-sectional study

**DOI:** 10.1186/s13104-017-2893-1

**Published:** 2017-11-10

**Authors:** Chatté Adawaye, Joseph Fokam, Erick Kamangu, Hamit Mahamat Alio, Aoudalkarim Moussa Chahad, Fabrice Susin, Ali Mahamat Moussa, Tchombou Hig-Zounet Bertin, Abdelsalam Tidjani, Dolores Vaira, Michel Moutschen

**Affiliations:** 1 Institut National Supérieur des Sciences et Techniques d’Abéché, Abéché, Chad; 2 Virology Laboratory, Chantal BIYA International Reference Centre for research on HIV/AIDS prevention and management, Yaoundé, Cameroon; 3 Department of Experimental Medicine and Surgery, Faculty of Medicine and Surgery, University of Rome Tor Vergata, Rome, Italy; 4 Faculty of Medicine and biomedical Sciences, University of Yaoundé I, Yaoundé, Cameroon; 5 National HIV Drug Resistance Working Group, Ministry of Public Health, Yaoundé, Cameroon; 6 Département des Sciences de Base, Faculté de Médecine, Université de Kinshasa, Kinshasa, Democratic Republic of the Congo; 7 Faculté des Sciences de la Santé Humaine/Hôpital Général de Référence Nationale, Ndjamena, Chad; 8 Laboratoire de Référence SIDA, CHU de Liège, Liège, Belgium; 9 Service des Maladies Infectieuses et Médecine Interne Générale, Centre Hospitalier et Universitaire de Liège, Liège, Belgium

**Keywords:** First-line antiretroviral therapy, Virological response, Drug resistance, HIV-1 subtypes, Adults, Chad

## Abstract

**Background:**

The national antiretroviral therapy in the Republic of Chad provides free of charge antiretroviral regimens and therapeutic monitoring for patients receiving antiretroviral therapy nationwide. For a successful programmatic uptake, these efforts merit to be supported by thorough assessments of antiretroviral therapy response and HIV-1 drug resistance surveillance, especially with risks of cross-resistance due to the gradual stavudine phasing out in such national settings. We therefore evaluated the virological response to antiretroviral therapy, HIV-1 drug resistance emergence and circulating HIV-1 clades in a Chad context. A cross-sectional and prospective study was conducted among 116 patients (41 [δ ± 6.87] years, 59% female) receiving first-line antiretroviral therapy for ≥ 6 months in Ndjamena, Chad, in 2011–2012, enrolled consecutively. To ensure accuracy, plasma viral load was concomitantly measured using *Abbott Real*-*Time* and *Cobas AmpliPrep/TaqMan (v2.0)*, and virological failure defined as ≥ 1000 HIV-1 RNA copies/ml. Plasma from patients experiencing virological failure were processed for sequencing of HIV-1 protease-reverse transcriptase using the *ANRS*-*AC.11* resistance testing protocol; drug resistant mutations were interpreted using the *ANRS*-*AC11* algorithm; and phylogenetic analysis was performed using *MEGA.v.6*.

**Results:**

Majority of patients was receiving zidovudine plus lamivudine plus nevirapine (46%), stavudine plus lamivudine plus nevirapine (41%) and tenofovir plus emtricitabine plus efavirenz (11%), for a median time-on-treatment of 5 [IQR 4–7] years. The rate of virological failure was 43% (50/116), with 86% (43/50) sequencing performance. Overall, 32% (37/116) patients presented ≥ one major drug resistant mutation(s), with 29% (34/116) to nucleos(t)ide reverse transcriptase inhibitors (67% [29/43] M184V/I, 30% [13/43] T215Y/F, 19% [8/43] V75A/F/I/L/M, 9% [4/43] K70P/R/W, 9% [4/43] K219E/N/Q and 5% [2/43] A62V); 86% (37/43) to non-nulceos(t)ide reverse transcriptase inhibitors (30% [13/43] K103N/S/E, 26% [11/43] Y181C/V/F/L, 2% [1/43] L100I, 2% [1/43] F227L, 2% [1/43] P225H); and 2% (1/43) to protease inhibitors (M46I, I54V, V82S). Six HIV-1 subtypes were found: 30% circulating recombinant form (CRF02_AG), 30% J, 16% G, 9% A, 9% D, 5% F.

**Conclusions:**

In Chad, almost half of patients are failing first-line antiretroviral therapy after 5 years, with considerable drug resistant mutations at failure. Absence of K65R supports the use of tenofovir-containing regimens as preferred first-line and as suitable drug for second-line combinations, in this setting with significant HIV-1 genetic diversity.

**Electronic supplementary material:**

The online version of this article (10.1186/s13104-017-2893-1) contains supplementary material, which is available to authorized users.

## Background

HIV/AIDS remains a major cause of death worldwide, and especially in sub-Saharan Africa (SSA) where over 71% of the global AIDS epidemic is concentrated in only 12% of the world population. In spite of a reduction in HIV-associated morbidity and mortality in SSA, about half of people living with HIV (PLHIV) still ignore their status, suggesting a potential growing burden of HIV in this region of the world [1–3].

Located in central Africa, Chad is a country with 3.3% of HIV prevalence in the sexually active population (i.e. 15–45 years) for a national population of 11.4 million inhabitants [4–6]. Interestingly, Chad is the fifth largest African country, partly bordered by Cameroon, a country known as the epicenter of HIV with a broad genetic diversity that includes several HIV-1 groups M, N, O and P, and HIV-2, as well as several subtypes and recombinants [7–11]. HIV-1 group-M predominates the molecular epidemiology in Chad (subtypes A, D, F, G, CRF01_AE, CRF02_AG and CRF11_cpx); few cases of group O have been reported while groups N and P, while HIV-2 have never been identified [10, 12, 13]. Exploring the extent of HIV diversity in Chad would therefore provide updates and related impact on the dynamics of national AIDS epidemics for relevant policy-making [14].

ART management and laboratory monitoring are effective and free-of-charge in the national AIDS program in Chad since 2007, with first-line regimens consisting of two nucleoside reverse transcriptase inhibitor (NRTI) and one non-NRTI (NNRTI). As per the World Health Organization (WHO) recommended guidelines, preferred first-line regimens since 2012 consist of “tenofovir (TDF), emtricitabine (FTC) and efavirenz (EFV)” or “zidovudine (AZT), lamivudine (3TC) and nevirapine (NVP)”; following phasing-out of Triomune due to lipodistrophy/lipoatrophy and peripheral neuropathy significantly associated with “stavudine” (d4T)-containing regimens [5, 8, 14]. However, at the moment of the study, viral load testing was implemented only at the national reference hospital laboratory. Viral load was mainly performed as needed, after treatment failure based on immunological and/or clinical parameters, thus indicating a limited accessibility to virological monitoring nationwide during the study period. Thus, mastering HIVDR profile in such context will help in predicting potential cross-resistance to currently used regimens [14, 15].

As first-line regimens used in Chad mainly consist of drugs with low-genetic barriers to resistance, risks of HIV-1 drug resistance (HIVDR) emergence are concerning. Of note, over 60% of ART failure was previously reported [14, 16], supporting the need for local HIVDR surveillance to sustain the effectiveness of first-line ART, to inform on the selection of active NRTI for second-line combinations, to generate evidence on the dynamics of HIV-1 genetic diversity and potential relevance on therapeutic response for patients receiving ART according to the current national treatment program [5, 6].

In this study, we sought to ascertain the rate of virological failure (VF), the level of drug resistance mutations and HIV-1 genetic diversity among people living with HIV (PLHIV) receiving first-line ART as per the Chadian AIDS program in N’Djamena.

## Methods

### Study design and population

A prospective and cross-sectional study was conducted in PLHIV receiving first-line ART at the National Reference General Hospital of N’djamena *(Hôpital Général de Référence National de Ndjaména)* in Chad, between 2011 and 2012. This hospital was selected as sentinel site based on its role as the national reference center in Chad, its long-term experience on ART and its technical capacity in providing reference laboratory monitoring for ARV management. Participants were eligible if: (a) receiving first-line ART for ≥ 6 months, (b) self-reported adherent to prescribed ARV medications, (c) registered and followed-up on ART at the study clinic, and (d) providing consent as study participants.

### Sampling method

A non-probability sampling was used, by which patients were conveniently enrolled based on accessibility throughout the study period.

Following informed consent, participants were interviewed and assessed for eligibility criteria, then enrolled as study participants if eligible.

Whole blood was collected in two EDTA tubes of 4 ml each, through venipuncture, and plasma was collected following centrifugation at 2000*g* for 10 min. Plasma aliquots of 1 ml were prepared and stored at − 80 °C.

### Measurement of TCD4 lymphocytes

To evaluate the stage of disease progression, CD4 T lymphocytes were measured for all patients at baseline and at 6 [± 2] months of ART, based on the fluorescent activated cell sorting approach, using the commercially available FACS Count as per the manufacturer’s instructions (Becton–Dickinson Immunocytometry Systems, USA).

### Measurement of plasma viral load

Plasma viral load (PVL) was performed at the AIDS Reference Laboratory of the University Health Center (*Laboratoire de Référence Sida du CHU*) in Liege, Belgium, using two different approaches purposely to ensure accuracy on these non-B viral populations: (1) COBAS^®^ AmpliPrep/CobasTaqman^®^ HIV-1 version 2.0 (v2.0) which is based on in vitro amplification of HIV-1 RNA from plasma with detection thresholds ranging from 20 (lower) to 10,000,000 (upper) RNA copies/ml designed specifically for HIV-1 groups M and O; (2) the Abbott RealTime HIV-1 test Ref 2G3190 which is based on in vitro amplification by RT-PCR for the quantification of HIV-1 in plasma. Both PVL tests were performed as per manufacturers’ instructions.

VF was defined as PVL ≥ 1000 HIV-1 RNA copies/ml; plasma samples from study participants experiencing VF were designated for HIV-1 sequencing for the detection of DRMs and for viral subtyping.

### RNA extraction, amplification and sequencing

#### RNA extraction

RNA extraction was performed on plasma using the QIAamp mini kit (*Qiagen, Courtaboeuf,* France), as per the manufacturer’s instructions. Briefly, 140 µl plasma samples stored at − 80 °C was extracted using lysis and wash buffers, followed by elution of 60 µl RNA.

#### Amplification and Sequencing reactions

The ANRS AC11 protocol was used for amplification and sequencing of the protease (PR) and reverse transcriptase (RT) regions of HIV-1, using GeneAmp PCR System 9700 thermal cycler [17]. Briefly, amplification was performed using Titan One tube RT-PCR Kit version 13 (Boehringer Mannheim, Manneheim, Germany), with a first-round PCR using primers amplifying 941 bp of RT (MJ3/MJ4) and 653 bp that encompasses the entire PR (5′Prot1/3′Prot1) region. Second-round (nested) PCR was performed with primers A35/NE(1)35 covering 731 bp of RT and primers 5′prot2/3′prot2 covering 507 bp that encompasses the entire PR. Alternative outer primers used for RT were RT18/RT21 and for PR 5′eprB/3′eprB, while alternative nested primers were RT1/RT4 for RT and 5′prB/3′prB for PR regions. Primer sequences are provided in Additional file 1.

Revelation of PCR products was done using 4% ethidium bromide agarose gel electrophoresis, with an expected size of 731 pb for RT and 507 for PR, including positive and negative controls alongside a molecular ladder (Tacklt ™ ΦX174 RF DNA/Hae III Fragment). Amplicons were purified PCR using NucleoFast^®^ 96 PCR (Macherey–Nagel).

As per the ANRS AC11 protocol, HIV-1 PR-RT was sequenced using overlapping primers by deoxyterminators [17]. Sequences were purified using resin Sephadex G-50, and identified following capillary electrophoresis on a “3730” genetic analyzer of Applied Biosystem (ABI).

#### Interpretation of HIV-1 drug resistance

Following the sequencing protocol used [17], HIV-1 DRMs were interpreted according to the ANRS AC11 algorithm (http://www.hivfrenchresistance.org/). Viruses with a mutant or a mixture of wild type and mutant, at an amino acid’s position, were considered to have the resistant variant. Patients were considered as harbouring wild type viruses if their viral load was < 1000 RNA copies/ml (virological success) or with a non-amplifiable sample.

#### HIV-1 phylogenetic analysis

Sequence alignment was done using CLUSTAL W version 1.7, then sequences were trimmed and gaps closed [18]. Phylogenetic inference was performed with MEGA version 6 [19], using Neighbor Joining with 1000 replicates and Kimura two-parameter [20, 21]. Subtypes were assigned for bootstrap ≥ 70% with a reference sequences from a pure HIV-1 subtype or recombinant strain obtained from Genbank (http://www.hiv.lanl.gov/).

### Statistical analysis

Data were processed using EPI INFO version 3.3.2. Chi square test was used for analysing categorical data on VF according to ART regimens, including 95% CI, with a *P* < 0.05 considered statistically significant. Spearman correlation was used for quantitative data on PVL results obtained from the two different instruments, with R ≥ 0.8 considered as a strong positive correlation.

## Results

### Profile of study participants

Overall, 116 PLHIV treated at the National Reference General Hospital of N’djamena were enrolled in the study, divided into 59% (68) female versus 41% (48) male. Mean age of these participants was 41 (± 6.87) years, min–max: 17–84 years (Additional file 2). Median CD4 was 248 [interquartile range (IQR) 145; 504] cells/mm^3^, min–max: 11–684 cells/mm^3^.

Among study participants, the median time-on-ART was 5 [IQR 4; 7] years. In terms of first-line drug regimens commonly prescribed at the study period, 87% (101/116) were receiving nevirapine (NVP) plus lamivudine (3TC) plus zidovudine (AZT) or stavudine (d4T), followed by 11% on a tenofovir-containing regimen (Table 1).

**Table 1:** Prescribed antiretroviral regimens

Treatment regimens	Number	Percentage
AZT+3TC+NVP	53	45.69
d4T+3TC+NVP	48	41.38
FTC+TDF+EFV	13	11.21
ABC+3TC+EFV	1	0.86
ABC+3TC+EFV	1	0.86
Total	116	100

*ABC* Abacavir, *AZT* Zidovudine, *3TC* lamuvidine, *EFV* efavirenz, *NVP* nevirapine, *d4T* stavudine, *TDF* tenofovir, *FTC* emtricitabine

#### Viral measurements and virological response to first-line ART

**Fig. 1:**
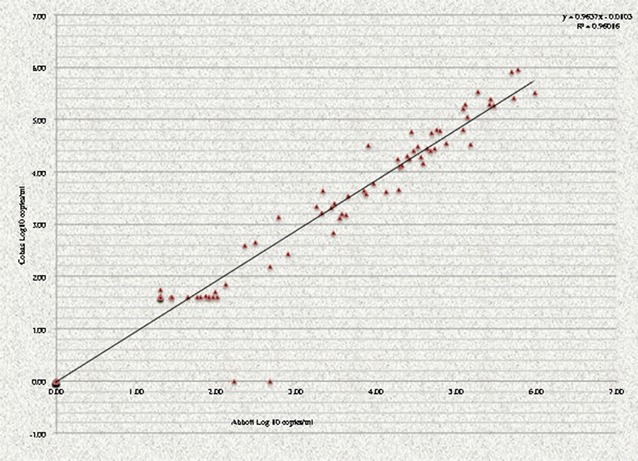
Correlation analysis between Abbott Real time HIV-1 and CobasTaqman^®^. Log10 represents the logarithm value from absolute numbers of plasma viral loads

All one hundred and sixteen study participants were tested for PVL using both described quantitation assays (*Cobas AmpliPrep/TaqMan*v2.0 and Abbott Real time HIV-1), and 109 samples yield comparable levels of HIV-1 RNA copies between both assays. A strong positive correlation (R^2^ = 0.96016) was observed between both PVL assays (Fig. 1), with only two samples reported  “*not detected*” with Abbott Real time HIV-1 and detected as low-levels (2.23 and 2.68 Log_10_ RNA copies/ml) viremia with *Cobas AmpliPrep/TaqMan* v2.0. Overall, both PVL assays accurately detect all patients with VF (≥ 1000 HIV-1 RNA copies/ml). Further characteristics of the two assays are provided in Additional file 3.

Out of 116 participants, 50 (43%) experienced VF. Between the two most prescribed regimens, VF was higher with d4T-containing (49% [24/49]) compared to other regimens (39% [26/67]), odd ratio (OR): 1.514 [95% CI 0.672–3.417), P = 0.274 (see Table 2).

**Table 2:** Virological failure per antiretroviral regimens

Treatment regimens	Patients per regimen	Virological failure per regimen, n (%)
AZT+3TC+NVP	53	19 (35.85%)
d4T+3TC+NVP	48	*23 (47.92%)*
FTC+TDF+EFV	13	6 (46.15%)
ABC+3TC+EFV	1	1 (100%)
ABC+3TC+EFV	1	1 (100%)

Italic value indicates the most prescribed antiretroviral regimen

*ABC* Abacavir, *AZT* Zidovudine, *3TC* lamuvidine, *EFV* efavirenz, *NVP* nevirapine, *d4T* stavudine, *TDF* tenofovir, *FTC* emtricitabine

The low number of participants on the other regimens could not allow a relevant statistical evaluation of response to ART.

### HIV-1 drug resistance mutations

#### Sequencing performance

All 50 samples from patients classified as VF (PVL ≥ 1000 HIV-1 RNA copies/ml) were processed for HIV-1 genotypic resistance testing (GRT), resulting to 86% (43/50) sequencing performance with the ANRS AC11 genotyping protocol [17].

#### HIV-1 mutations associated with resistance to reverse transcriptase inhibitors

The overall rate of patients with DRMs to reverse transcriptase inhibitors was 32% (37/116), including both nucleoside and non-nucleoside inhibitors.

**Fig. 2:**
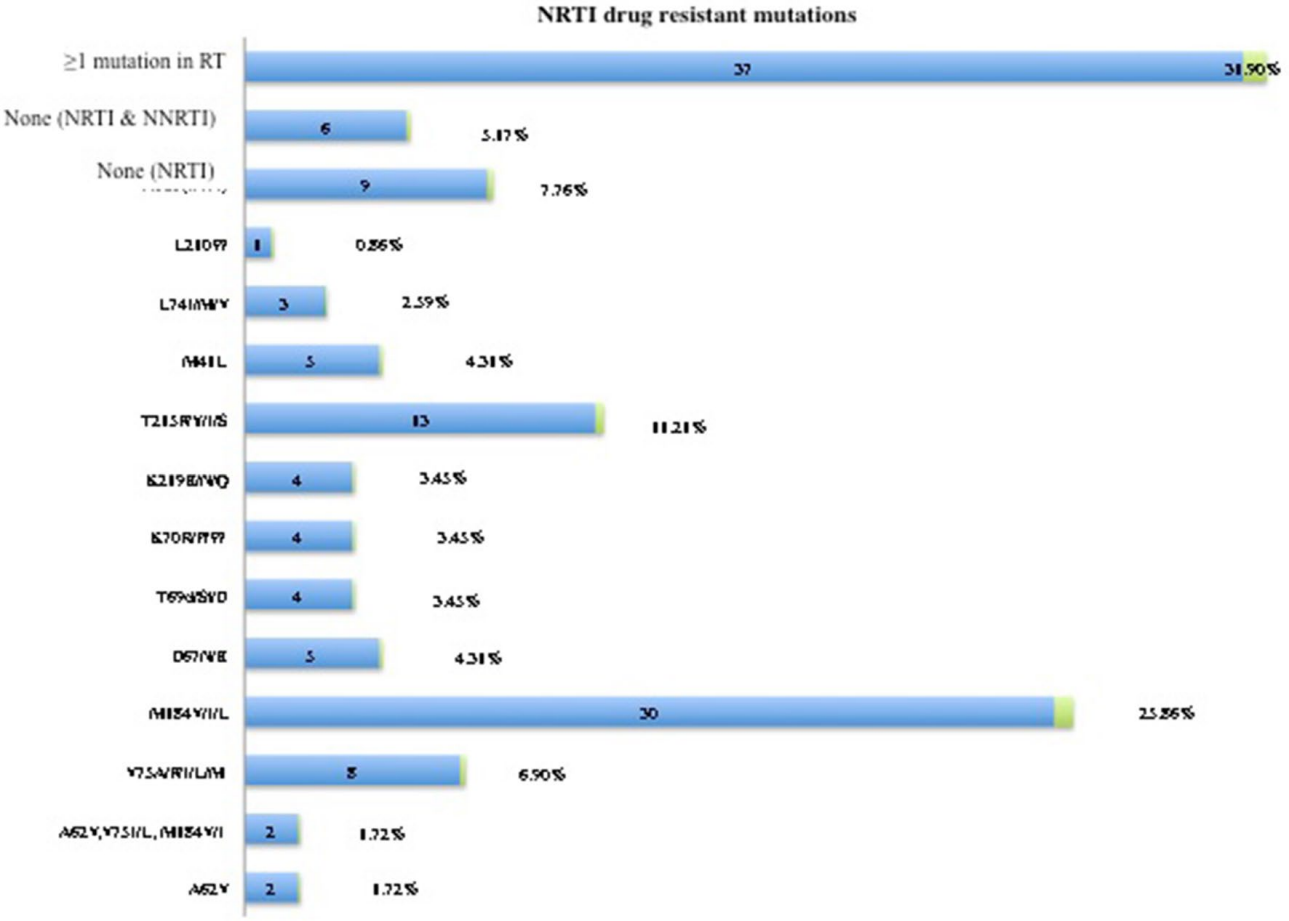
Prevalence of NRTI DRMs. *NRTI* nucleoside reverse transcriptase inhibitors, *RT* reverse transcriptase, *DRMs* drug resistant mutations

Thirty-four participants had at least one major DRM to nucleoside reverse transcriptase inhibitors (NRTIs), resulting to 29% (34/116) prevalence of NRTI DRMs in the entire study population. Out of the 43 sequences generated, the most prevalent DRMs were: (67% [29/43] M184V/I, 30% [13/43] T215Y/F, 19% [8/43] V75A/F/I/L/M, 9% [4/43] K70P/R/W, 9% [4/43] K219E/N/Q and 5% [2/43] A62V, followed by other DRMs observed at low rates (Fig. 2).

**Fig. 3:**
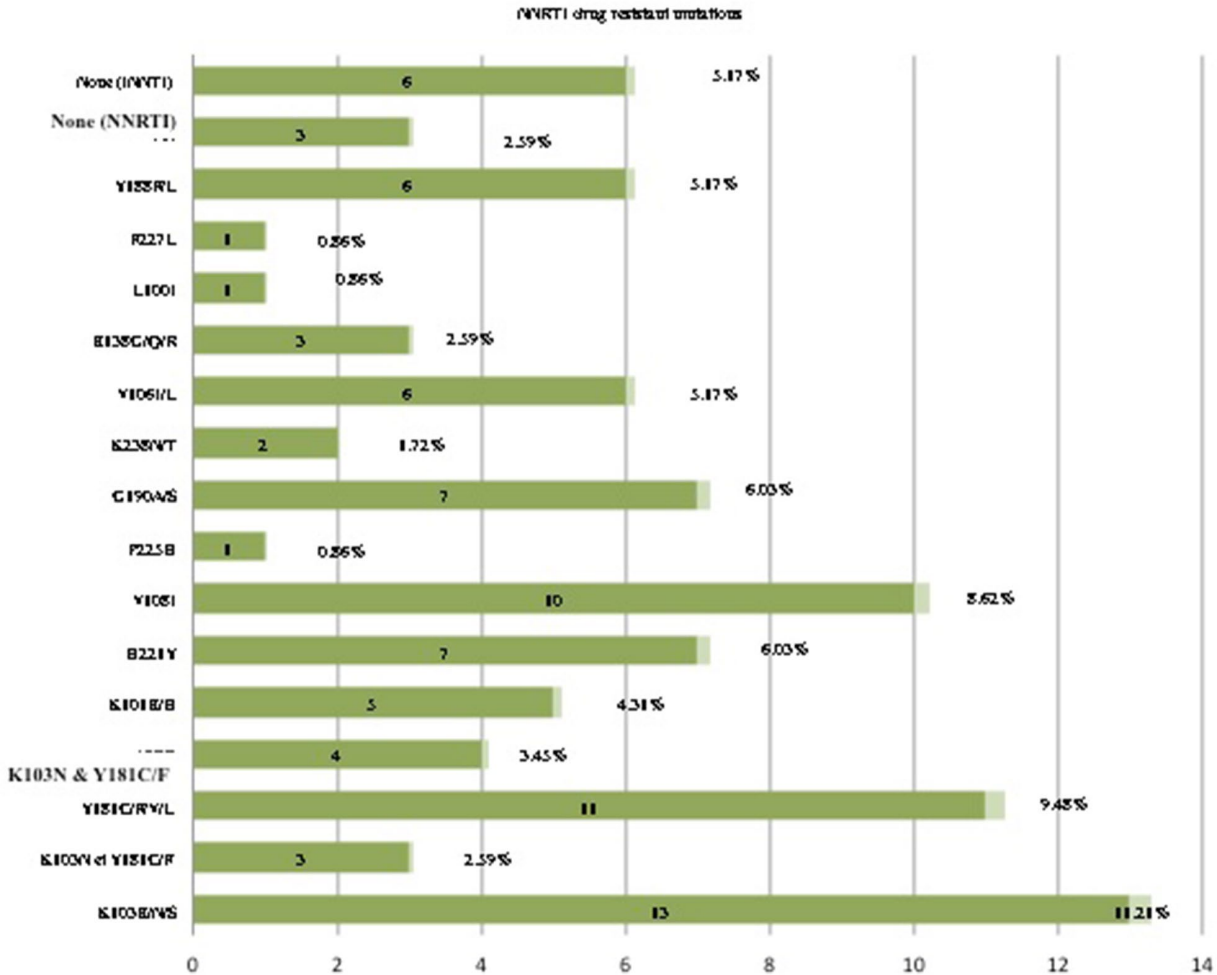
Prevalence of NNRTI DRMs. *NNRTI* non-nucleoside reverse transcriptase inhibitors, *DRMs* drug resistant mutations

Thirty-seven participants had at least one major DRM to non-nucleoside reverse transcriptase inhibitors (NNRTIs), resulting to 32% (37/116) prevalence in the entire study population. Out of the 43 sequences generated, the most prevalent DRMs were: 30% [13/43] K103N/S/E, 26% [11/43] Y181C/V/F/L, 2% [1/43] L100I, 2% [1/43] F227L and 2% [1/43] P225H, followed by other DRMs observed at lower rates. Of note, Y181C/F and K103N were observed concomitantly in three (7%) cases. Thus, in the entire study population, NNRTI DRMs exhibit 28% (32/116) and 21% (24/116) high-level resistance to nevirapine and efavirenz respectively (Fig. 3).

OF note, six (12%) patients, classified as VF, were reported without any DRMs, suggesting possible poor adherence in spite of the self-reported adherence registered prior to enrolment. The list of genotypic scores associated to each RTI is provided in Additional file 4.

#### HIV-1 mutations associated with resistance to protease inhibitors

Only one patient (< 1%) was reported with major DRMs to protease inhibitors (PI/r), among which M46I, I54V, and V82S, indicating either an event of transmitted PI-associated DRMs or unknown past-exposure to PIs. Minor mutations found were mainly polymorphisms: K20I/M (21), L10I/V (15), L90W (1), L76S (1), N88D (1), V11I (1), V32L (1) and G48R (1).

#### HIV-1 genetic diversity

**Fig. 4:**
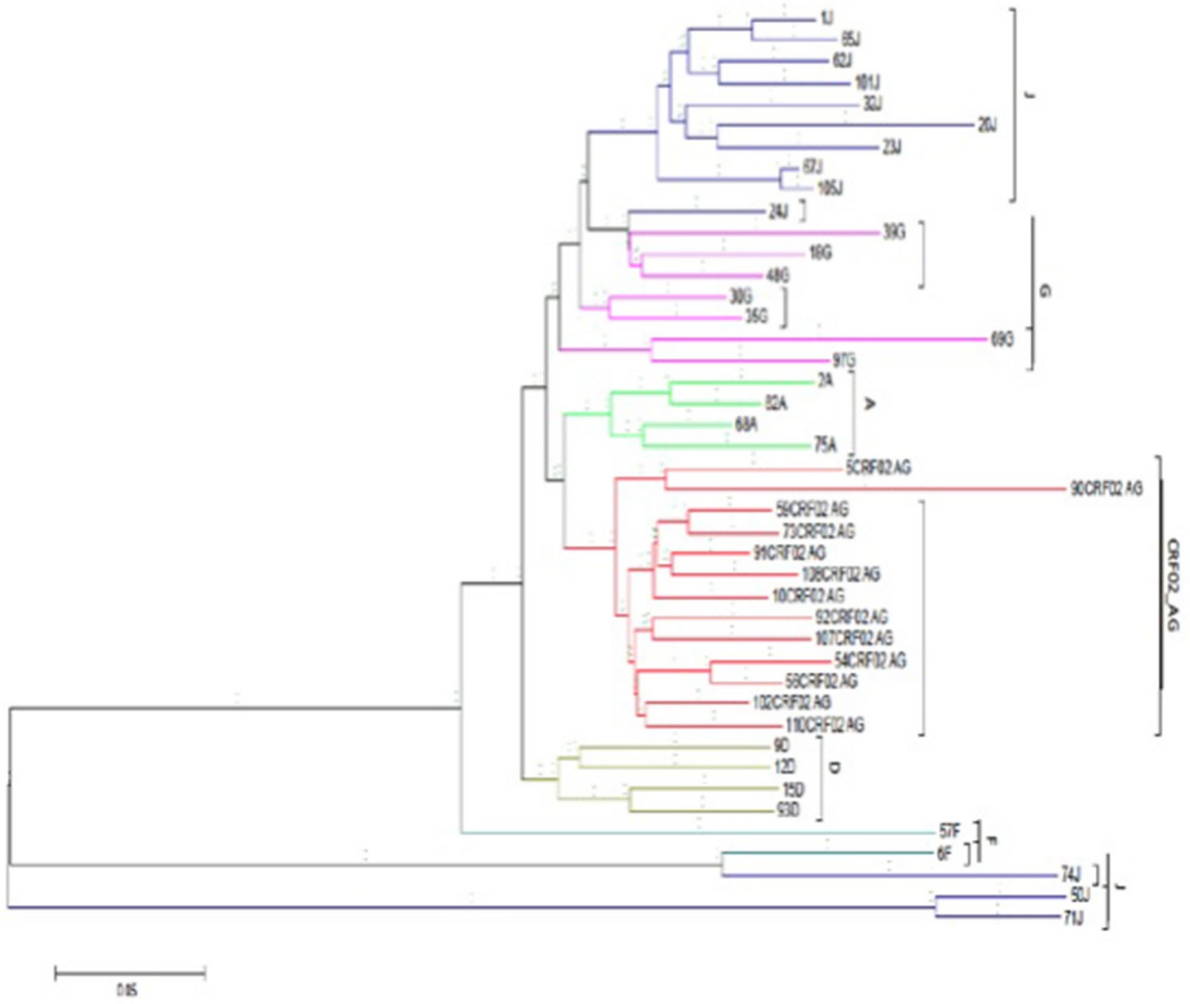
Phylogenetic tree. The evolutionary history was inferred using the Neighbor-Joining method in the protease-reverse transcriptase regions (Saitou and Nei [20]). The tree is drawn to scale, with branch lengths in the same units as those of the evolutionary distances used to infer the phylogenetic tree. The evolutionary distances were computed using the Kimura 2-parameter method (Kimura [21]) and are in the units of the number of base substitutions per site. The analysis involved 43 query nucleotide sequences. All positions containing gaps and missing data were eliminated. Evolutionary analyses were conducted in MEGA6 (Tamura et al. [19])

The 43 protease-RT sequences generated clustered within six clades (five pure subtypes and one recombinant). Of note, the two equally most prevailing were subtype J (30%) and CRF02_AG (30%), followed by subtypes G (16%), A (9%), D (9%) and F (5%), as shown in Fig. 4.

## Discussion

The success of combination ART has remarkably changed the paradigm in the AIDS epidemics globally. However, such achievements could rapidly be hampered in settings where ART is mainly based on drugs with low genetic barrier to resistance [14, 22, 23]. Since PLHIV in Chad are treated with RTI- or PI/r-containing regimens, evaluating the virological response, acquired HIVDR and circulating strains are of great programmatic asset in sustaining ART performance in a medium-long run [6, 15, 16].

Our findings indicated that, about 5 years after ART initiation, almost half of PLHIV on first-line NRTI/NNRTI regimens in Chad would be experiencing VF, suggesting close virological monitoring is needed in the country ART program [15, 24–27]. More importantly, as higher (though non-significant) rate of VF was observed with d4T-containing regimens (49% vs. 39% for other regimens, OR = 1.514), added to the known adverse effects of this, our findings support effective phasing-out of d4T from first-line ART regimens from this setting, while monitoring for cross-resistance to AZT and other NRTIs commonly used in first- and second-line combinations [6, 14, 28].

The high rate of VF in our cohort could be attributed to infrequent PVL measurement in 2011–2012 due to the centralized system, thus resulting to delayed testing and suboptimal monitoring. At the moment of the study, VL was possible only at the National Reference General Hospital of N’djamena, the only facility nationwide whereby VL is routinely offered since 2005 to date. Presently, the *Entre*-*aide 92* team from Paris is assessing the feasibility of using GeneXpert for point-of-care VL in the cities of Moundou and Am Timan, in order to scale-up access to VL in Chad [29]. Therefore, although based on a limited sample, our findings could be representative of the general country situation.

Amongst those experiencing VF, relatively lower rates of NRTI and NNRTI mutations were found, possibly due to suboptimal adherence, in the frame of poor ART monitoring. This calls for an improved adherence support to enhance and sustain viral suppression in the country [29].

For an accurate evaluation of virological response, a strong positive correlation in PVL was reported for samples tested concomitantly with two different platforms, which in turns confirms assay reliability in settings where non-B HIV-1 prevails [23]. Of note, the two discrepant cases were patients with low-level PVL (< 1000 copies/ml), both clinically classified as virological success [3], or blips (transient rebound in PVL returning to undetectable levels with adherence) [8].

As levels of acquired HIVDR are higher to NNRTIs (32%) compared to NRTIs (29%), conferring high-level resistance to NVP and EFV (mainly due to K103N and Y181C), NRTIs are more prone for use in second-line combinations in such ART program [30–32]. Of note, K65R, the main DRM to TDF was not detected, supporting TDF-containing NRTIs as preferred combinations to second-line LPV/r, as globally recommended [3, 14]. Moreover, our data also support using the combination of TDF plus (FTC or 3TC) plus EFV as the most prominently active first-line regimen in such RLS, at the moment [9, 14]. Even though 3TC and FTC are highly hampered by M184V (~ 29%) in the overall study population, the ability of this mutation in decreasing viral replicative fitness and in improving susceptibility to thymidine analogs suggest maintaining these drugs (3TC and FTC) within current treatment guidelines [3, 9, 33, 34]. Very low resistance to ABC also favors this drug as a suitable NRTI substitute for second-line combination, especially in case of counter indication to TDF [3, 35, 36]. Most importantly, the very low-level of HIVDR to PI/r confirms the suitability to LPV/r, ATV/r and other PI/r as backbone for second-line ART, in combination with potentially active NTRIs [3, 6, 9, 13].

A higher prevalence of CRF02_AG was found in Chad as compared to previous findings [10, 12], possibly due the ability of AG-recombinant in being more infectious but with less cytopathic effect [37–40]. Our small sample (43 sequences) therefore calls for enlarged molecular epidemiology studies to better understand the HIV-1 genetic diversity, its evolution overtime and related clinical relevance in the country [41].

A study limitation would be the “self-reported” adherence, making it difficult to verify the reliability of recalls. Phasing out of D4T may suggest not representativeness of the data on current ART regimens. However, D4T is the same drug class with AZT (analogs of TAMs), and several patients have been exposed to these drugs. Our findings are therefore useful for all patients with past-exposure to the drug class of TAMs [42, 43].

Complementary studies are needed to ascertain response after switch from first- to second-line [9, 44, 45], to evaluate the feasibility of point-of-care resistance testing designed with commonly found mutations (M184V/I, T215mutants, K103N, Y181C) for greater cost-effectiveness [46, 47], and monitoring HIVDR early warning indicators [48].

### Programmatic implications for the ART program in Chad

Our findings address issues that could be translated into policies. Of note, in addition to the need for closed viral load monitoring for timely detection of treatment failures, the high failure rate of ART in Chad also indicates a rapid switch of patients from first- to second-line ART regimens. Rapid switch to second-line ART lead to increased costs of ARVs provision for the national HIV program in Chad, thus representing a major programmatic challenge for such RLS. Interestingly, the unusually low rate of DRMs, developing after significant periods of treatment failure, suggests suboptimal adherence, thereby underscores the usefulness to closely monitor ART adherence and the need for confirming VF (i.e. a second viral load after counselling and adherence support) before deciding on ART switch. Such measure would help clinicians in distinguishing elevated viremia due to non-adherence, thereby limiting unnecessary switch to second-line regimens while saving related-costs in the national ART program of Chad.

## Conclusions

About half of Chadian PLHIV experienced VF by medium-term after ART initiation, suggesting closed virological monitoring using commonly available commercial assays. For successful phasing-out of d4T, DRMs supports using current WHO-recommended TDF plus TFC (or 3TC) plus EFV as preferred first-line regimen in Chad, while LPV/r is potentially active as second-line backbone, alongside “TDF” or “ABC”, in association with NRTIs. The predominant rate of recombinant AG is a quest for further investigation within the sub-region.

## Additional files

**Additional file 1.** PCR and sequencing Primers (ANRS AC 11). The primer sequences are designed for amplification and sequencing reactions of protease and reverse transcriptase regions.

**Additional file 2.** Study participants by age range. The table details the study population by range age from 17 to over 60 years old, divided by male and female.

**Additional file 3.** Characteristics of assays used for plasma viral load. The table reports the minimal and maximal values, as well as mean and median of viral loads, obtained on one hand with Cobas and on the other hand with Abbott.

**Additional file 4.** Level of resistance to reverse transcriptase inhibitors. The table presents the proportion of patients with levels of genotypic susceptibility score following the Stanford algorithm.

## Abbreviations

3TC: lamivudine
ABI: applied biosystem
AIDS: acquired immunodeficiency syndrome
ANRS: agence nationale pour la recherche sur le SIDA et les hépatitis virales
ARV: antiretroviral
ART: antiretroviral therapy
AZT: zidovudine
CHU: centre hospitalier universitaire
D4T: stavudine
DRM: drug resistance mutation
EDTA: ethidium diamine tetra acetate
EFV: efavirenz
FACS: fluorescent activated cell sorting
FTC: emtricitabine
HIVDR: HIV-1 drug resistance
HIV: human immunodeficiency virus
MEGA: molecular evolutionary genetics analysis
NNRTI: non-nucleoside reverse transcriptase inhibitor
NRTI: nucleoside reverse transcriptase inhibitor
NVP: nevirapine
PCR: polymerase chain reaction
PLHIV: people living with HIV
PVL: plasma viral load
PR: protease
RLS: resource-limited setting
RNA: ribonucleic acid
RT: reverse transcriptase
RT-PCR: reverse-transcriptase polymerase chain reaction
SSA: sub-Saharan Africa
TAM: thymidine analog mutation
TDF: tenofovir
WHO: World Health Organization
