# Supplementary material for: Virological response, HIV-1 drug resistance mutations and genetic diversity among patients on first-line antiretroviral therapy in N’Djamena, Chad: findings from a cross-sectional study
Source: BMC Res Notes. 2017 Nov 10;10:589. doi: 10.1186/s13104-017-2893-1 (PMC5681824; doi:10.1186/s13104-017-2893-1)
Supplement: Supplementary file 1 — Additional file 1. PCR and sequencing Primers (ANRS AC 11). The primer sequences are designed for amplification and sequencing reactions of protease and reverse transcriptase regions. [file 13104_2017_2893_MOESM1_ESM.docx]

**Additional file 1: PCR and sequencing Primers (ANRS AC 11)**

• Reverse Transcriptase (codons 20-240):

Outer primers

MJ3: 5’-AGTAGGACCTACACCTGTCA-3’ (2480 to 2499)

MJ4: 5’-CTGTTAGTGCTTTGGTTCCTCT-3’ (3399 to 3420)

Inner primers (amplification: 798 bp fragment)

A(35): 5’-TTGGTTGCACTTTAAATTTTCCCATTAGTCCTATT-3’ (2530 to 2558)

NE1(35): 5’-CCTACTAACTTCTGTATGTCATTGACAGTCCAGCT-3’ (3300 to 3334)

Sequencing primers

A(20): 5’-ATTTTCCCATTAGTCCTATT-3’

alternatively :HIV55 : 5’ CCAAAAGTTAAACAATGGCCATTGACAGA 3’

NE1(20): 5’-ATGTCATTGACAGTCCAGCT-3’

Outer primers alternatively used for RT (codons 30- 225)

RT18: 5’- GGA AAC CAA AAA TGA TAG GGG GAA TTG GAG G -3’

RT21: 5’- CTG TAT TTC TGC TAT TAA GTC TTT TGA TGG G-3’

Inner and sequencing primers:

RT1: 5’- CCA AAA GTT AAA CAA TGG CCA TTG ACA GA -3’

RT4: 5’- AGT TCA TAA CCC ATC CAA AG –3’

• Protease:

Outer primers:

5’ prot 1: 5’-TAATTTTTTAGGGAAGATCTGGCCTTCC-3’ (2082 to 2109)

3’ prot 1 5’-GCAAATACTGGAGTATTGTATGGATTTTCAGG-3’ (2703 to 2734);

Inner (amplification: 507 bp fragment) and sequencing primers

5’ prot 2: 5’-TCAGAGCAGACCAGAGCCAACAGCCCCA-3’ (2136 to 2163)

3’ prot 2: 5’-AATGCTTTTATTTTTTCTTCTGTCAATGGC-3’ (2621 to 2650);

Other sets of primers that can be used alternatively for protease:

Outer primers:

5’ eprB: 5’ AGA GCT TCA GGT TTG GGG 3’

3’ eprB: 5’ GCC ATC CAT TCC TGG CTT 3’

Inner and sequencing primers:

5’ prB: 5’ GAA GCA GGA GCC GAT AGA CA 3’

3’ prB: 3’ ACT GGT ACA GTT TCA ATA GG 3’
