# Supplementary material for: Virological response, HIV-1 drug resistance mutations and genetic diversity among patients on first-line antiretroviral therapy in N’Djamena, Chad: findings from a cross-sectional study
Source: BMC Res Notes. 2017 Nov 10;10:589. doi: 10.1186/s13104-017-2893-1 (PMC5681824; doi:10.1186/s13104-017-2893-1)
Supplement: Supplementary file 2 — Additional file 2. Study participants by age range. The table details the study population by range age from 17 to over 60 years old, divided by male and female. [file 13104_2017_2893_MOESM2_ESM.docx]

**Additional file 2: Study participants by age range**

| Age range | | | | | | | | | | |
| --- | --- | --- | --- | --- | --- | --- | --- | --- | --- | --- |
|  | 17-24 | 25-30 | 31-35 | 36-40 | 41-45 | 46-50 | 51-55 | 56-60 | >60 | Total |
| M | 2 | 7 | 5 | 5 | 11 | 10 | 2 | 3 | 3 | 48 |
| F | 1 | 0 | 15 | 25 | 14 | 8 | 4 | 0 | 1 | 68 |
| Total | 3 | 7 | 20 | 30 | 25 | 18 | 6 | 3 | 4 | 116 |
