# Supplementary material for: Virological response, HIV-1 drug resistance mutations and genetic diversity among patients on first-line antiretroviral therapy in N’Djamena, Chad: findings from a cross-sectional study
Source: BMC Res Notes. 2017 Nov 10;10:589. doi: 10.1186/s13104-017-2893-1 (PMC5681824; doi:10.1186/s13104-017-2893-1)
Supplement: Supplementary file 3 — Additional file 3. Characteristics of assays used for plasma viral load. The table reports the minimal and maximal values, as well as mean and median of viral loads, obtained on one hand with Cobas and on the other hand with Abbott. [file 13104_2017_2893_MOESM3_ESM.docx]

**Additional file 3: Characteristics of assays used for plasma viral load**

|  | Cobas | | Abbott | |
| --- | --- | --- | --- | --- |
|  | Copies/ml | Log10 | Copies/ml | Log10 |
| Minimum PVL | 0.00 | 0 | 0.00 | 0 |
| Maximum PVL | 966.000 | 5,98 | 885.196 | 5.95 |
| Mean PVL | 43.301 | 4.64 | 43.460.96 | 4.6 |
| Median PVL | 85 | 1.93 | 51 | 1.7 |
