# Supplementary material for: Virological response, HIV-1 drug resistance mutations and genetic diversity among patients on first-line antiretroviral therapy in N’Djamena, Chad: findings from a cross-sectional study
Source: BMC Res Notes. 2017 Nov 10;10:589. doi: 10.1186/s13104-017-2893-1 (PMC5681824; doi:10.1186/s13104-017-2893-1)
Supplement: Supplementary file 4 — Additional file 4. Level of resistance to reverse transcriptase inhibitors. The table presents the proportion of patients with levels of genotypic susceptibility score following the Stanford algorithm. [file 13104_2017_2893_MOESM4_ESM.docx]

**Additional file 4: Level of resistance to reverse transcriptase inhibitors**

| ARV | High-level reresistance | Score | Intermediate resistance | Score | Susceptible | Score | Potential low-level resistance | Score | Low-level resistance | Score |
| --- | --- | --- | --- | --- | --- | --- | --- | --- | --- | --- |
| 3TC | 29 (67.5%) | 60-110 | 00 (00.0%) | 0 | 13 (30.2%) | 0-5 | 00 (00.0%) | 0 | 00 (00.0%) | 0 |
| AZT | 06 (13.9%) | 65-155 | 09 (20.9%) | 30-45 | 27 62.8%) | -10-0 | 00 (00.0%) | 0 | 00 (00.0%) | 0 |
| D4T | 06 (13.9%) | 65-155 | 10 (23.3%) | 30-45 | 26 (60.5%) | -10-0 | 00 (00.0%) | 0 | 00 (00.0%) | 0 |
| EFV | 24 (55.8%) | 60-110 | 10 (23.3%) | 30-55 | 07 (16.3%) | 0 | 02 (04.7%) | 10 | 00 (00.0%) | 0 |
| NVP | 32 (74.4%) | 60-150 | 02 (04.7%) | 40-55 | 07 (16.3%) | 0 | 00 (00.0%) | 0 | 00 (00.0%) | 15 |
| FTC | 30 (69.8%) | 60-110 | 00 (00.0%) | 0 | 13 (30.2%) | 0-5 | 00 (00.0%) | 0 | 00 (00.0%) | 0 |
| TDF | 01 (02.3%) | 110 | 03 (06.9%) | 30-35 | 33 (76.7%) | -10-5 | 01 (2.3%) | 10 | 04 (09.3%) | 15-25 |
| ABC | 04 (09.3%) | 100-135 | 09 (20.9%) | 30-45 | 09 (20.9%) | 0 | 00 (00.0%) | 0 | 20 (46.51%) | 15-25 |

**Legend:** 3TC (lamivudine); ABC (abacavir); AZT (zidovudine); D4T (stavudine); EFV (efavirenz); NVP (nevirapine); TDF (tenofovir).
